# The Ratio of Estimated Average Glucose to Fasting Plasma Glucose Level Is Superior to Glycated Albumin, Hemoglobin A1c, Fructosamine, and GA/A1c Ratio for Assessing **β**-Cell Function in Childhood Diabetes

**DOI:** 10.1155/2014/370790

**Published:** 2014-06-10

**Authors:** Ji Eun Lee, Ji Woo Lee, Tatsuyoshi Fujii, Noriyoshi Fujii, Jong Weon Choi

**Affiliations:** ^1^Department of Pediatrics, College of Medicine, Inha University, Incheon, Republic of Korea; ^2^School of Medicine, University of Tsukuba, Ibaraki, Japan; ^3^Department of Electrical Engineering and Bioscience, Waseda University, Tokyo, Japan; ^4^Department of Laboratory Medicine, Inha University Hospital, College of Medicine, Inha University, 7-206 Shinheung-dong 3-Ga Jung-gu, Incheon 400-711, Republic of Korea

## Abstract

*Objective*. This study investigated the use of the estimated average glucose to fasting plasma glucose ratio (eAG/fPG ratio) to screen for **β**-cell function in pediatric diabetes. *Methods*. Glycated hemoglobin (HbA1c), glycated albumin (GA), fructosamine, insulin, and C-peptide levels were measured. The ratio of GA to HbA1c (GA/A1c ratio) was calculated, and the homeostasis model assessment of **β**-cell function (HOMA-**β**) was determined. *Results*. Median values of C-peptide, insulin, and HOMA-**β** levels were significantly higher in patients with an increased eAG/fPG ratio than in those with a decreased eAG/fPG ratio. C-peptide and HOMA-**β** levels were more closely correlated with the eAG/fPG ratio than with GA, HbA1c, the GA/A1c ratio, and fructosamine. In contrast, body mass index was significantly associated with GA, GA/A1c ratio, and fructosamine, but not with the eAG/fPG ratio and HbA1c levels. To test the diagnostic accuracies of the eAG/fPG ratio for identifying HOMA-**β** > 30.0% in patients with type 2 diabetes, the area under the ROC curve of the eAG/fPG ratio was significantly larger than that of the GA/A1c ratio [0.877 (95% CI, 0.780–0.942) versus 0.775 (95% CI, 0.664–0.865), *P* = 0.039]. *Conclusions*. A measurement of the eAG/fPG ratio may provide helpful information for assessing **β**-cell function in pediatric patients with diabetes.

## 1. Introduction

The major fraction of glycated hemoglobin, HbA1c, has been widely used to assess the long-term glycemic control and the risk for the development of complications in diabetes. Measurement of HbA1c has recently been recommended for diagnosing diabetes. However, HbA1c is affected by a variety of conditions, such as hemolytic anemia, chronic renal failure, and the presence of variant hemoglobins [1, 2]. HbA1c levels are underestimated in patients with poor glycemic control because survival of erythrocytes is shortened under hyperglycemic conditions [3].

Fructosamine was previously introduced in an index of glycemic control for two weeks past, measuring the amount of total glycosylated protein in serum. Fructosamine is not affected by anemia or variant hemoglobins but the level is influenced by the concentrations of serum protein, bilirubin, uric acid, and low molecular weight substances coexisting in the blood [4, 5].

Glycated albumin (GA) is an alternative marker reflecting shorter-term glycemic control, which is influenced less by disorders of hemoglobin metabolism. Since albumin is glycosylated at approximately 10 times the rate of hemoglobin, GA is sensitive to the change of blood glucose levels [6]. In addition, GA is a reliable parameter to evaluate neonatal diabetes, hemodialysis patients, and gestational diabetes [7–9]. However, GA is also influenced by the pathologic condition affecting albumin metabolism, such as nephrotic syndrome, liver cirrhosis, or thyroid dysfunction [10].

Recently, the ratio of GA to HbA1c (GA/A1c ratio) has been reported to reflect postprandial glucose excursion and relates to **β**-cell function in both type 1 and type 2 diabetes [11]. However, physiologic variables, such as age or body mass index (BMI), make the GA/A1c ratio a little unpredictable in clinical practice [12].

Few studies have closely examined the relationship between endogenous insulin production and the ratio of estimated average glucose to fasting plasma glucose levels (eAG/fPG ratio) in pediatric diabetes. The present study investigated the usefulness of the new parameter, the eAG/fPG ratio, to screen for **β**-cell function in children and adolescents with diabetes mellitus (DM), particularly comparing the GA/A1c ratio and the three glycated proteins.

## 2. Materials and Methods

### 2.1. Subject Populations

A total of 137 patients with type 1 diabetes (T1DM, *n* = 62) and type 2 diabetes (T2DM, *n* = 75) were studied, who visited the Department of Pediatrics at Inha University Hospital from March 2011 to December 2013. Their ages ranged from 3 to 18 (median age, 13 years), and 63 patients were males (46.0%). All the patients fit the criteria for the diagnosis of diabetes by the American Diabetes Association [13]. Differentiating T1DM from T2DM was based on laboratory tests, patient characteristics, and history as follows: C-peptide level (fasting plasma C-peptide < 0.6 ng/mL, postprandial plasma C-peptide < 1.5 ng/mL, and urinary C-peptide secretion < 10 *μ*g/24 hours), presence of insulin or islet autoantibodies, and a history of diabetic ketoacidosis [13–15].

Clinical and demographic data were collected retrospectively from a review of medical records, including the duration of DM and history of treatment. Patients with anemia (*n* = 2), liver disease (*n* = 1), and recent infection (*n* = 2) were excluded from the study. Subjects with missing values (*n* = 3), history of operation (*n* = 1), and administration of anti-inflammatory drugs (*n* = 2) were also excluded from analysis. This study was approved by the Institutional Review Board of Inha University Hospital.

### 2.2. Measurement of Parameters

The following parameters were measured: glycated proteins (HbA1c, GA, and fructosamine), glucose levels (fPG and postprandial plasma glucose), glycemic index ratio (GA/A1c ratio and eAG/fPG ratio), **β**-cell function (C-peptide and insulin), anthropometric parameters (height, weight, and BMI), and glycemic index-associated parameters (serum albumin, hemoglobin, and serum creatinine). Homeostasis model assessment of **β**-cell function (HOMA-**β**) and insulin resistance (HOMA-IR) was determined by a HOMA2 calculator to assess the basal **β**-cell function and insulin resistance in patients with T2DM [16]. BMI was calculated as weight in kilograms divided by the square of the height in meters. Blood specimens were collected prior to insulin treatment.

Serum insulin and C-peptide levels were measured by an immunoradiometric assay with insulin and C-peptide IRMA kits, respectively (Beckman Coulter, Fullerton, CA, USA). The HbA1c fraction was measured by high-performance liquid chromatography with EDTA-anticoagulated blood using a G7 Glycohemoglobin Analyzer (Tosoh Bioscience, South San Francisco, CA, USA). Serum GA levels were analyzed by a turbidimetric immunoassay using a Hitachi 7180 analyzer (Hitachi, Tokyo, Japan). Fructosamine was assayed using a colorimetric assay on a Modular P Roche system (Roche Diagnostics GmbH, Mannheim, Germany). Plasma glucose levels, serum albumin, and serum creatinine levels were analyzed with a chemical analyzer (Hitachi 7600; Hitachi, Tokyo, Japan). Hemoglobin levels were measured with an automated analyzer (ADVIA 120; Siemens, Forchheim, Germany) using EDTA-anticoagulated blood.

### 2.3. Calculation of eAG/fPG Ratio

The eAG was calculated using the following equation: eAG (mmol/L) = 1.59 × HbA1c (%) − 2.59 [17]. The eAG/fPG ratio was computed using the following formula: eAG/fPG ratio = eAG level (mmol/L)/fPG level (mmol/L). The estimated glomerular filtration rate (eGFR) was determined by the Schwartz formula [18]: eGFR (mL/min/1.73 m^2^) = proportionality constants × height (cm)/serum creatinine level (mg/dL). The proportionality constants were 0.55 to 0.70: children at ages of 2–12 (0.55), girls at ages of 13–21 (0.55), and boys at ages of 13–21 (0.70), respectively.

Patients were categorized into 2 groups: T1DM (*n* = 62) and T2DM (*n* = 75). Patients with T2DM were further stratified into 2 groups, based on median values of the glycemic index ratio: the GA/A1c ratio <2.21 (*n* = 37) and ≥2.21 (*n* = 38); the eAG/fPG ratio <1.48 (*n* = 37) and ≥1.48 (*n* = 38).

### 2.4. Statistical Analysis

Data were presented as mean ± standard deviation (SD) if normally distributed and as median (range) if nonnormally distributed. Normality of the data distribution was tested by a Kolmogorov-Smirnov's one-sample test. Categorical variables were expressed as frequencies and proportions. A Mann-Whitney *U* test and a Student's* t*-test were used to analyze data between the two groups. A multivariate regression analysis of the eGA/fPG ratio, the GA/A1c ratio, GA, HbA1c, and fructosamine was conducted as a dependent variable in patients with T2DM. Adjustments for age, BMI, duration of diabetes, serum creatinine, and C-peptide levels were performed as independent variables.

A receiver operating characteristics (ROC) curve was analyzed to compare the diagnostic accuracy of the eAG/fPG ratio and the GA/A1c ratio to identify HOMA-**β** > 30.0% in T2DM. This figure was based on the cutoff value for the 25th percentile for HOMA-**β** of the 75 patients with T2DM included in this study. Accuracy was established by using the 95% confidence interval (CI) for the difference rate between the two parameters.

To estimate the optimal decision point of the C-peptide level for differentiating T1DM from T2DM, a ROC curve was generated using the GA/A1c ratio, based on the nine threshold values of the C-peptide levels. Data analysis was conducted using SPSS software (version 14.0, SPSS Inc., Chicago, IL, USA). All *P* values <0.05 were considered statistically significant.

## 3. Results

### 3.1. eAG/fPG Ratio versus GA/A1c Ratio

The baseline characteristics of subject populations in relation to T1DM and T2DM are summarized in Table 1. There were no significant differences in median age, eGFR, serum albumin, and hemoglobin levels between patients with T1DM and those with T2DM. However, median values of HbA1c, GA, and fructosamine levels were significantly higher in T1DM than in T2DM (8.7%, 24.1%, and 415 *μ*mol/L versus 7.9%, 14.8%, and 281 *μ*mol/L, resp., *P* < 0.05). Median levels of the GA/A1c ratio and the eAG/fPG ratio in patients with T1DM were 2.82 and 1.03, which were significantly different from the values of the parameters in those with T2DM (2.21 and 1.48, resp., *P* < 0.05) (Table 1).

As shown in Table 2, C-peptide, insulin, and HOMA-**β** levels in patients with an eAG/fPG ratio ≥1.48 were 3.95 ng/mL, 24.6 U/mL, and 154.2%, which significantly exceeded the levels of the corresponding parameters in those with an eAG/fPG <1.48 (2.75 ng/mL, 12.0 U/mL, and 63.5%, resp., *P* < 0.05). Similarly, C-peptide, insulin, and HOMA-**β** levels had increased to a significantly greater extent in patients with a GA/A1c ratio <2.21 than in those with a GA/A1c ratio ≥2.21 (4.35 ng/mL, 33.4 U/mL, and 158.9% versus 2.86 ng/mL, 12.2 U/mL, and 64.9%, resp., *P* < 0.05).

### 3.2. Multivariate Regression Analysis

In a multivariate analysis adjusted for the independent variables, the HOMA-**β** level was more closely correlated with the eAG/fPG ratio than with the GA/A1c ratio, GA, HbA1c, and fructosamine (*r* = 0.472 versus *r* = −0.410, *r* = −0.385, *r* = −0.402, and *r* = −0.391, resp., *P* < 0.001). The C-peptide levels were significantly associated with the eAG/fPG ratio, but not with the GA/A1c ratio, GA, HbA1c, and fructosamine levels. In contrast, BMI was significantly linked to the GA/A1c ratio, GA, and fructosamine but was not linked to the eAG/fPG ratio and HbA1c levels. There were significant correlations between the duration of diabetes and the values of GA, HbA1c, the GA/A1c ratio, and fructosamine; however, no significant correlation was observed between the eAG/fPG ratio and the duration of diabetes (Table 3).

**Figure 1:**
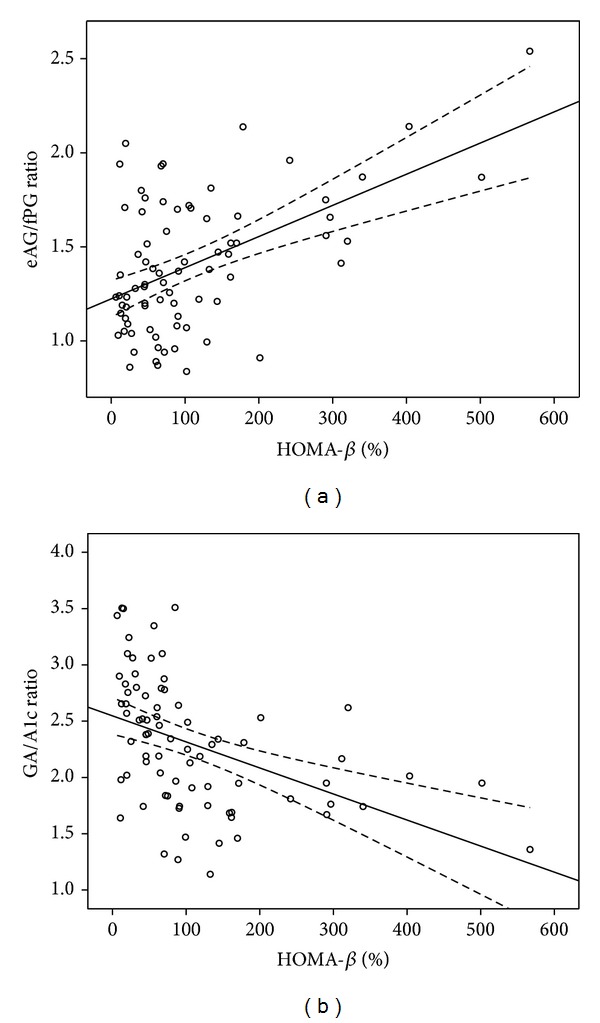
Scatter plots showing the correlation between HOMA-**β** and the values of the eAG/fPG ratio (a) and the GA/A1c ratio (b) in T2DM. HOMA-**β** correlates positively with the eAG/fPG ratio (*y* = 0.0017*x* + 1.224; *r* ^2^ = 0.267; *P* < 0.001) but correlates inversely with the GA/A1c ratio (*y* = −0.0023*x* + 2.548; *r* ^2^ = 0.201; *P* < 0.001). HOMA-**β**, homeostasis model assessment of **β**-cell function; eAG/fPG ratio, the ratio of estimated average glucose to fasting plasma glucose; GA/A1c ratio, the ratio of glycated albumin to HbA1c level.

A linear regression analysis between HOMA-**β** and the values of the eAG/fPG ratio and the GA/A1c ratio in T2DM is displayed in Figure 1. Scatter plots showed that HOMA-**β** had a positive correlation with the eAG/fPG ratio but had an inverse correlation with the GA/A1c ratio. The correlation coefficient between HOMA-**β** and the eAG/fPG ratio (*r* ^2^ = 0.267, *P* < 0.001) was higher than that between HOMA-**β** and the GA/A1c ratio (*r* ^2^ = 0.201, *P* < 0.001).

### 3.3. ROC Curve Analysis

**Figure 2:**
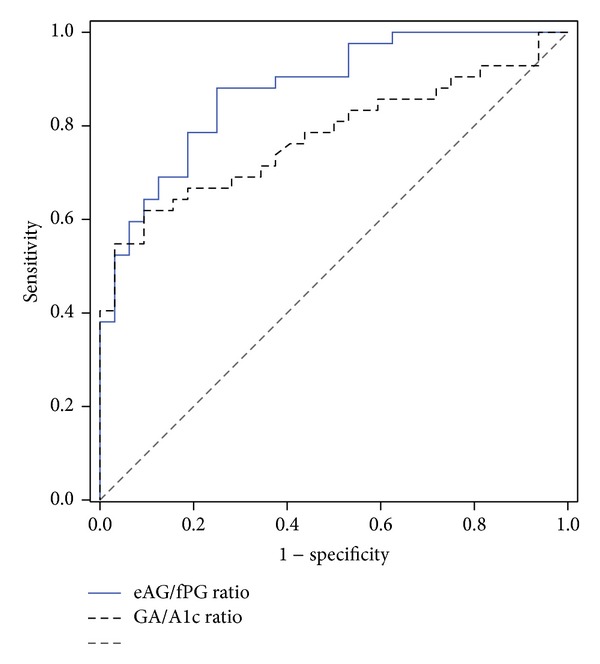
Comparison of ROC curves (eAG/fPG ratio versus GA/A1c ratio) for identifying HOMA-*β* > 30.0% in patients with T2DM. The eAG/fPG ratio (AUC, 0.877; 95% CI, 0.780–0.942; sensitivity 88.1%; and specificity 75.2% at the optimal cutoff of 1.16), the GA/A1c ratio (AUC, 0.775; 95% CI, 0.664–0.865; sensitivity 61.5%; and specificity 90.6% at the optimal cutoff of 2.53), and difference of AUCs between the two ratios (AUC, 0.102; 95% CI, −0.042 to 0.243; *P* = 0.039).

The diagnostic accuracies of the eAG/fPG ratio and the GA/A1c ratio to identify HOMA-*β* > 30.0% in patients with T2DM were investigated. In a ROC curve analysis, the area under the curve (AUC) of the eAG/fPG ratio was significantly larger than that of the GA/A1c ratio [0.877 95% CI, 0.780–0.942 versus 0.775 (95% CI, 0.664–0.865), *P* = 0.039]. The cutoff points of the eAG/fPG ratio and the GA/A1c ratio to detect HOMA-**β** >30.0% were 1.16 and 2.53, where the sensitivity and specificity of the eAG/fPG ratio were 88.1% and 75.2% and those of the GA/A1c ratio were 61.5% and 90.6%, respectively (Figure 2).

**Figure 3:**
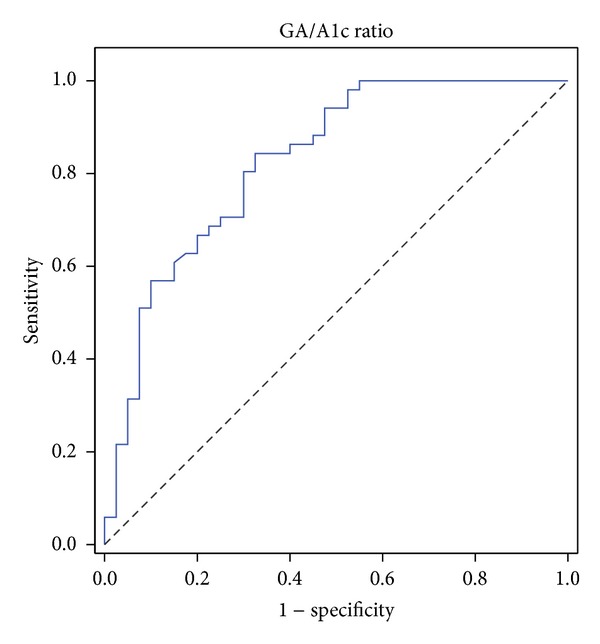
An example of a ROC curve by the GA/A1c ratio, based on C-peptide level (1.0 ng/mL) in pediatric diabetes. Area under the curve was calculated for the GA/A1c ratio (AUC, 0.826; 95% CI, 0.722–0.918; sensitivity 84.3%; and specificity 67.5% at the optimal cutoff of 2.50).

To estimate the optimal cutoff of the C-peptide level to distinguish T1DM from T2DM, patients were stratified into nine groups according to C-peptide levels (0.1 ng/mL to 2.5 ng/mL). On the basis of each level of C-peptide, ROC curves were generated using the GA/A1c ratio. Of 9 cutoffs for C-peptide levels, 5 (55.6%) demonstrated the AUCs >0.75, and the rest revealed the AUCs > 0.65. An AUC based on a C-peptide level of 1.0 ng/mL was 0.826 (95% CI, 0.722–0.918), which was larger than any other AUC (Table 4). The ROC curve, which was generated on the basis of a C-peptide level of 1.0 ng/mL, was illustrated in Figure 3, showing sensitivity (84.3%) and specificity (67.5%) at the optimal cutoff (2.50) of the GA/A1c ratio.

## 4. Discussion

In this study, a new parameter of the eAG/fPG ratio was determined using HbA1c-derived average glucose and the present plasma glucose levels. The parameter was used to screen for **β**-cell function in patients with T2DM, comparing with the well-known parameters, such as the GA/A1c ratio, HbA1c, GA, and fructosamine. Median values of C-peptide, insulin, and HOMA-**β** levels were significantly higher in patients with an increased eAG/fPG ratio than in those with a decreased eAG/fPG ratio. The eAG/fPG ratio more strongly correlated with HOMA-**β** and C-peptide levels than did the GA/A1c ratio, HbA1c, GA, and fructosamine. These results suggest that the new parameter may accurately reflect endogenous insulin secretion in pediatric patients with T2DM.

In our study, median levels of the GA/A1c ratio in patients with T1DM were significantly above the values of the GA/A1c ratio in those with T2DM. Our data are in general agreement with the previous results of Yoshiuchi et al. [19], which demonstrated that the GA/A1c ratio was significantly higher in patients with T1DM than in those with T2DM.

Koga et al. [20] reported that HOMA-**β** had a significant inverse correlation with the GA/A1c ratio, suggesting relatively higher serum GA to HbA1c levels in patients with decreased insulin production. In contrast, Huh et al. [21] reported that the direct effect of HOMA-**β** on the GA/A1c ratio was not significant in diabetic patients. In our study, the GA/A1c ratio showed a negative correlation with the HOMA-**β** level but showed no significant correlations with HOMA-IR and C-peptide levels. Our data support the results of Kim et al. [16], which disclosed that the GA/A1c ratio was significantly associated with HOMA-**β** and BMI, but not with HOMA-IR. These discrepancies may reflect the differences in patient populations, the severity of disease, the glucose tolerance status, and the degree of obesity among the studies.

Obesity is known to be negatively associated with GA and the GA/A1c ratio. However, conflicting data on the relationship of the obesity versus GA and serum albumin levels have been reported. A group of investigators reported that obesity-related chronic inflammation plays a role in decreasing GA levels [22]. Several researchers have suggested that declined serum albumin levels in obese patients may contribute to the inverse correlation between obesity and GA [23].

Conversely, Nishimura et al. [24] reported that obese children showed a high serum albumin concentration in comparison with nonobese children, but Koga et al. [22] found that no correlation existed between obesity and serum albumin levels. In our study, BMI had a significant inverse correlation with the GA/A1c ratio, GA, and fructosamine. These observations suggest that the GA/A1c ratio has a limitation, which is affected by the severity of obesity of the subject populations in the various studies.

Because glycemic variability is responsible for the development of diabetic complications, multiple measures are conducted to evaluate the magnitude of glycemic excursion [25]. Alternative methods of reporting blood glucose levels have been proposed. The eAG infers an average glucose levels from the HbA1c concentrations, which may efficiently inform patients of their glycemic control [26]. In the present study, the eAG/fPG ratio was compared with the GA/A1c ratio to assess **β**-cell function in patients with T2DM. To the best of our knowledge, there has been no study in the literature to date focusing on the relationship between the eAG/fPG ratio and **β**-cell function in childhood diabetes.

As the eAG level has a linear functional relation to HbA1c level, the meaning of the eAG/fPG ratio is similar to that of HbA1c/fPG ratio. However, in this study we presented the data for the eAG/fPG ratio instead of the HbA1c/fPG ratio. Because the eAG/fPG ratio is yielded from the homogeneous parameters with the same units, the values for the eAG/fPG ratio can be interpreted with ease. On the other hand, the HbA1c/fPG ratio is the rate for the heterogeneous variables with different units, which is ultimately generating a complicated unit (%·L/mmoL).

After adjusting for age, BMI, duration of disease, and serum creatinine levels, the regression analysis consistently demonstrated a significant relationship between the eAG/fPG ratio and C-peptide levels. However, no significant correlation was observed between the GA/A1c ratio and C-peptide levels, after adjusting for the corresponding parameters. Furthermore, the diagnostic accuracy of the eAG/fPG ratio was significantly higher than that of the GA/A1c ratio for identifying HOMA-**β** >30.0% in T2DM. These results suggest that the eAG/fPG ratio more exactly represents endogenous insulin production than the GA/A1c ratio. Our results also suggest that the eAG/fPG ratio is less affected by obesity, compared to the GA/A1c ratio.

Huh et al. [21] found that the factors influencing the GA/A1c ratio were different according to glucose tolerance status: the GA/A1c ratio cannot be an accurate index of glycemic control in normal glucose tolerance (HbA1c ≤ 5.6%), although it may be a significant index in diabetes. It is assumed that the GA/A1c ratio is affected by glucose tolerance status as well as obesity in diabetic patients. These findings may be derived from the characteristics of two parameters: the GA/A1c ratio was calculated using the two-glycated proteins, which are synthesized via a relatively long-term glycated process; however, the eAG/fPG was calculated using the current glucose level and the mean plasma glucose level. This is a likely explanation as to why there were no significant correlations between the eAG/fPG ratio and the duration of diabetes in our subject populations, contrary to the findings of significant correlation between the GA/A1c ratio and the duration of diabetes.

Increased insulin resistance and **β**-cell dysfunction are important contributing factors to the pathophysiology of type 2 diabetes [27]. C-peptides, which are released from pancreatic **β**-cells during the biosynthesis of insulin, are an indicator of endogenous insulin production [28]. HOMA is a computational method for assessing **β**-cell function and insulin resistance and is widely used to assess the insulin sensitivity and resistance as a surrogate index [29, 30]. In our study, HOMA-**β** had more strongly correlated with GA than with HbA1c and fructosamine. Our data corroborate partly previous reports where GA and the GA/A1c ratios are significantly correlated with insulin secretory function in type 2 diabetic patients [16].

Differentiation between T1DM and T2DM has important implications for therapeutic decisions. In the overweight adolescent, differentiating T1DM from T2DM may be difficult. In children without autoantibodies, the use of plasma C-peptide levels has been recommended but the interpretation of such measurements is controversial [31]. For instance, various criteria for C-peptide levels have been used to determine T1DM: fasting C-peptide level < 0.5 ng/mL [16] or fasting C-peptide level < 0.6 ng/mL [14].

In the present study, to test the optimal cutoff point of C-peptide level for differentiating T1DM from T2DM, a ROC curve analysis was conducted using the GA/A1c ratio for the nine threshold values of C-peptide levels. The GA/A1c ratio showed a fairly good performance to distinguish T1DM from T2DM in combination with most cutoffs of C-peptide levels in childhood diabetes. The largest AUC was generated when a C-peptide level of 1.0 ng/mL was applied: AUC was gradually increased with the threshold values, reached the peak at the level of 1.0 ng/mL, and fell to the initial value. The C-peptide level of 1.0 ng/mL may be considered as another cutoff point to distinguish T1DM from T2DM in pediatric diabetes.

There are several limitations of this study. The study was confined to pediatric patients with diabetes. The number of subjects of this pilot study was too small to analyze the data in more detail. Therefore, we could not definitely determine the significance of the eAG/fPG ratio in adult patients with diabetes. Despite these limitations of our study, we believe that the eAG/fPG ratio is helpful for assessing **β**-cell function, at least in pediatric diabetics who had undergone only the fasting plasma glucose and HbA1c tests.

## 5. Conclusions

This study shows that the eAG/fPG ratio is more closely associated with C-peptide and HOMA-**β** levels than the GA/HbA1c ratio. Diagnostic accuracy of the eAG/fPG ratio was superior to that of the GA/A1c ratio to detect HOMA-**β** > 30.0% in T2DM, suggesting that the eAG/fPG ratio has a more significant implication with endogenous insulin secretion than the GA/HbA1c ratio. Measurement of the eAG/fPG ratio in conjunction with the GA/A1c ratio may offer additional benefits for monitoring **β**-cell function in pediatric patients with diabetes. Further studies are needed in larger populations of subjects, especially in adult diabetics, for the validation of the new parameter.

## Figures and Tables

**Table 1:** Baseline characteristics of the subject populations included in this study.

	Pediatric diabetes	
	Type 1 diabetes	Type 2 diabetes
Number of subjects	62	75
Age (year)	13 (3–17)	13 (9–18)
Gender (male, %)	29 (46.8)	34 (45.3)
Duration of diabetes (years)	2.3 (0.1–12.5)	0.8 (0.2–5.9)^a^
Height (cm)	149.5 ± 28.4	162.1 ± 10.2^a^
Weight (kg)	47.1 ± 15.2	70.6 ± 18.9^a^
BMI (kg/m^2^)	19.3 ± 4.5	26.5 ± 5.1^a^
Glycation index		
HbA1c (%)	8.7 (6.2–16.3)	7.9 (5.9–14.3)^a^
Glycated albumin (%)	24.1 (15.0–61.8)	14.8 (9.2–39.4)^a^
Fructosamine (µmol/L)	415 (263–749)	281 (194–605)^a^
Glucose levels		
Fasting plasma glucose (mmol/L)	9.6 (7.5–23.7)	7.5 (7.2–16.4)^a^
PP2hrs (mmol/L)	15.4 (9.4–28.6)	12.9 (9.1–36.2)^a^
*β*-Cell function		
C-peptide (ng/mL)	0.03 (0.01–0.59)	3.15 (0.82–14.4)^a^
Insulin (U/mL)	3.6 (1.0–9.7)	18.2 (7.5–58.3)^a^
HOMA-*β* (%)	NA	102.1 (12.9–753.5)
Insulin resistance		
HOMA-IR	NA	2.8 (0.8–7.3)
Glycemic index ratio		
GA/A1c ratio	2.82 (1.74–4.09)	2.21 (1.18–3.52)^a^
eAG/fPG ratio	1.03 (0.59–3.29)	1.48 (0.84–2.14)^a^
Glycation index-associated parameters		
Serum creatinine (mg/dL)	0.74 ± 0.14	0.78 ± 0.12
eGFR (mL/min/1.73 m^2^)	119.6 ± 16.8	127.0 ± 14.3
Serum albumin (g/dL)	4.38 ± 0.42	4.61 ± 0.35
Hemoglobin (g/dL)	13.6 ± 1.2	14.0 ± 1.8

Data are expressed as mean ± SD or median (range).

^a^Statistically significant (*P* < 0.05), versus type 1 diabetes, computed by a Mann-Whitney *U* test and a Student's *t*-test.

BMI, body mass index; HOMA-*β*, homeostasis model assessment of *β*-cell function; HOMA-IR, homeostasis model assessment of insulin resistance; PP2hrs, postprandial 2 hours; eAG/fPG ratio, the ratio of estimated average glucose to fasting plasma glucose; GA/A1c ratio, the ratio of glycated albumin to HbA1c level; eGFR, estimated glomerular filtration rate; NA, not applicable.

**Table 2:** C-peptide, HOMA-*β*, and HOMA-IR levels according to the median values of the GA/A1c ratio and the eAG/fPG ratio in patients with T2DM.

	Type 2 diabetes			
	GA/A1c ratio		eAG/fPG ratio	
	<2.21	≥2.21	<1.48	≥1.48
Number of subjects	37	38	37	38
Age (years)	13 (10–18)	13 (9–16)	12 (9–17)	13 (9–18)
BMI (kg/m^2^)	26.2 (13.4–39.2)	22.1 (15.8–29.6)^a^	23.2 (13.4–34.2)	25.1 (17.1–39.2)
Duration of diabetes (years)	1 (0–6.2)	0.3 (0–4.6)^a^	0.5 (0–4.3)	0.8 (0–6.2)
Fasting plasma glucose (mmol/L)	7.4 (7.2–12.1)	8.4 (7.4–16.4)^a^	8.2 (7.3–16.4)	7.4 (7.2–13.2)^b^
HbA1c (%)	6.7 (5.9–11.6)	8.1 (6.4–14.3)^a^	8.3 (6.2–14.3)	6.6 (5.9–11.4)^b^
Glycated albumin (%)	12.3 (9.2–30.7)	19.9 (12.5–39.4)^a^	19.1 (10.2–39.4)	12.4 (9.2–32.5)^b^
C-peptide (ng/mL)	4.35 (2.06–14.6)	2.86 (0.82–4.52)^a^	2.75 (0.82–5.47)	3.95 (2.12–14.6)^b^
Insulin (U/mL)	33.4 (14.2–58.3)	12.2 (5.37–24.8)^a^	12.0 (5.37–23.1)	24.6 (16.1–58.3)^b^
HOMA-*β* (%)	158.9 (91.1–753.5)	64.9 (12.9–79)^a^	63.5 (12.9–161.6)	154.2 (70.2–753.5)^b^
HOMA-IR	3.7 (1.4–7.2)	2.5 (0.8–4.7)	2.7 (0.8–4.9)	3.1 (1.6–7.2)

Data are expressed as median (range).

^a,b^Statistically significant (*P* < 0.05), versus groups with a GA/A1c ratio (<2.21) and an eAG/fPG ratio (<1.48), respectively, computed by a Mann-Whitney *U* test.

BMI, body mass index; HOMA-*β*, homeostasis model assessment of *β*-cell function; HOMA-IR, homeostasis model assessment of insulin resistance; eAG/fPG ratio, the ratio of estimated average glucose to fasting plasma glucose; GA/A1c ratio, the ratio of glycated albumin to HbA1c level.

**Table 3:** Multivariate regression analysis of the eGA/fPG ratio, the GA/A1c ratio, glycated albumin, HbA1c, and fructosamine as a respective dependent variable in patients with T2DM.

Variables	eAG/fPG ratio		GA/A1c ratio		Glycated albumin		HbA1c		Fructosamine	
	Model 1	Model 2	Model 1	Model 2	Model 1	Model 2	Model 1	Model 2	Model 1	Model 2
HOMA-*β*	0.517 (<0.001)	0.472 (<0.001)	−0.448 (<0.001)	−0.410 (<0.001)	−0.477 (<0.001)	−0.385 (<0.001)	−0.436 (<0.001)	−0.402 (<0.001)	−0.462 (<0.001)	−0.391 (<0.001)
Age (years)		0.066 (0.814)		0.141 (0.580)		0.063 (0.803)		−0.035 (0.889)		0.074 (0.766)
BMI (kg/m^2^)		0.164 (0.495)		−0.660 (<0.001)		−0.465 (<0.001)		−0.133 (0.537)		−0.538 (<0.001)
Duration of diabetes (years)		−0.191 (0.476)		−0.514 (<0.001)		−0.697 (<0.001)		−0.680 (<0.001)		−0.558 (<0.001)
Serum creatinine (mg/dL)		0.145 (0.501)		−0.123 (0.598)		0.108 (0.638)		0.315 (0.186)		0.169 (0.551)
C-peptide (ng/mL)		0.358 (0.041)		−0.106 (0.594)		−0.128 (0.531)		−0.119 (0.566)		−0.065 (0.791)

Correlations between the glycated indices and the various independent variables are expressed as standard *β* (*P* value). Model 1: unadjusted; model 2: adjusted for age, BMI, duration of diabetes, serum creatinine, and fasting C-peptide levels.

**Table 4:** Estimated cutoff point for C-peptide level to differentiate T1DM from T2DM using a ROC curve in childhood diabetes.

Cutoffs for C-peptide levels (ng/mL)	Area under ROC curve by GA/A1c ratio		
	AUC	95% confidence interval	*P* values
0.1	0.674	0.556–0.791	0.022
0.2	0.675	0.555–0.795	0.021
0.3	0.685	0.565–0.804	0.012
0.6	0.757	0.645–0.868	<0.001
1.0	0.826	0.722–0.918	<0.001
1.5	0.805	0.693–0.920	<0.001
1.8	0.779	0.652–0.905	<0.001
2.0	0.778	0.651–0.903	<0.001
2.5	0.691	0.625–0.889	<0.001
